# Potential reduction in healthcare carbon footprint by autonomous artificial intelligence

**DOI:** 10.1038/s41746-022-00605-w

**Published:** 2022-05-12

**Authors:** Risa M. Wolf, Michael D. Abramoff, Roomasa Channa, Chris Tava, Warren Clarida, Harold P. Lehmann

**Affiliations:** 1 Department of Pediatric Endocrinology, Johns Hopkins University School of Medicine, Baltimore, MD USA; 2 Department of Ophthalmology, University of Iowa, Iowa City, IA USA; 3 Digital Diagnostics, Coralville, IA USA; 4 Department of Ophthalmology, University of Wisconsin Madison, Madison, WI USA; 5 Department of Health Informatics, Johns Hopkins University School of Medicine, Baltimore, MD USA

**Keywords:** Environmental health, Population screening

## Abstract

Healthcare is a large contributor to greenhouse gas (GHG) emissions around the world, given current power generation mix. Telemedicine, with its reduced travel for providers and patients, has been proposed to reduce emissions. Artificial intelligence (AI), and especially autonomous AI, where the medical decision is made without human oversight, has the potential to further reduce healthcare GHG emissions, but concerns have also been expressed about GHG emissions from digital technology, and AI training and inference. In a real-world example, we compared the marginal GHG contribution of an encounter performed by an autonomous AI to that of an in-person specialist encounter. Results show that an 80% reduction may be achievable, and we conclude that autonomous AI has the potential to reduce healthcare GHG emissions.

## Introduction

There are efforts worldwide to reduce greenhouse gas (GHG) emissions, as they adversely affect the environment and human health^1^. Based on present, real world, mix of energy production methods (i.e., lacking renewables, nuclear, or other low or zero-carbon energy production), up to 10% of all annual GHG emissions may be due to healthcare processes and services^2^. The combined emissions of healthcare in the US, Canada, Australia, and the UK have been estimated to be ~7.5 × 10^14^ grams (g) of carbon dioxide equivalents (CO_2_-eq) annually^3^. These include energy use at the healthcare facility, including heating, ventilation and air conditioning, as well as its construction, provider, personnel and patient travel, training of physicians and other healthcare workers, healthcare technology and systems usage, pharmaceuticals and medical devices, and cleaning and disposables^3^.

Digital health technology, and specifically telemedicine or telehealth, has the potential to improve patient care, population health, reduce the per capita cost of healthcare, and improve the experience of providing care^4^. During the recent pandemic, these advantages became more apparent, as shown recently by Cortez et al.^5^. Importantly in this context, by reducing patient, physician and personnel travel, telemedicine also has the potential to substantially lower GHG emissions from travel energy use^6–11^.

Another digital health technology, artificial intelligence (AI), also has the potential to improve the quality, access, and experience of healthcare^12,13^. Such AI systems perform tasks intended to mimic human cognitive capabilities. These anthropomorphic AI systems are not explicitly programmed, and instead learn from data to perform highly cognitive tasks, such as those typically performed by trained healthcare professionals. As such, these AI systems have the potential to equalize access to healthcare for underserved populations, while improving care quality, at both the level of the individual patient and the population, at reduced cost for patient, payor, and society^14,15^. Initially, most healthcare AI systems were assistive, in other words, they assisted the physician or other provider in making medical decisions, but the ultimate authority for the clinical decision remained with the physician^16^. While such assistive AI may save time or effort on the part of the provider, it may also be additive to GHG emissions.

On the other hand, autonomous AI, where the medical decision is made by the AI system without human oversight, avoids the additive GHG emissions, and is infinitely scalable, with the potential to address healthcare disparities, improve patient outcomes, and patient and physician satisfaction^17–21^. The introduction of autonomous AI in the healthcare system has the potential to limit GHG emissions, and may even exceed the carbon emission reductions that resulted from telemedicine.

The goal of this study is to estimate the potential GHG emission reduction, if any, that can be achieved by the deployment of autonomous AI for point of care diagnostics.

## Healthcare and digital technology GHG emission estimation

Multiple studies have analyzed GHG emissions, typically quantified as carbon dioxide release, resulting from specific healthcare processes, such as cataract surgery, anesthesia, and critical care as a means to determine where emissions can be reduced^22–24^. Generally, the largest fraction of emissions was due to energy use, including transportation of patients, providers, and staff to and from the facility^10,11^. A study measuring transportation associated GHG emissions at a large U.S. regional health system demonstrated significant reductions in GHG over all outpatient visits, as the use of telemedicine increased: from 8 × 10^3^ g CO_2_-eq per visit to 4 × 10^3^ g CO_2_-eq per visit^25^. The authors went further in accounting for the potential increase in emissions related to use of computer equipment and internet for telemedicine visits, estimating that there would be a net increase of 5 × 10^7^ g CO_2_-eq for telemedicine encounters, compared to 8.7 × 10^9^ g CO_2_-eq reduction from patient travel avoided^25^. Similarly, a telemedicine program in Catalan (Spain) showed a significant reduction in emissions over the course of a year by avoiding more than 9000 in-person visits, and reducing travel time, fuel use, and carbon emissions related to patient travel^10^. Modeling this out across the US, expansion of telemedicine programs nationwide has the potential to reduce GHG emissions by 2 × 10^11 ^g CO_2_-eq annually^11^.

As mentioned above, while most outpatient GHG analyses, including the aforementioned studies, primarily consider patient travel-related emissions, they do not typically account for provider and personnel travel emissions, which is likely to be reduced^9^.

On the other hand, the use of digital technology itself, contributes to GHG emissions^26^. For example, a widely cited study based on data from 2015, estimated that digital technology is responsible for ~5 × 10^17 ^g CO_2_-eq emissions^27^. The training of a single AI system may create up to 5 × 10^8^ g CO_2_-eq emissions^28^, though inference—the calculation of a single output—may be only a fraction thereof^29^, and newer AI algorithms have shown improved GHG emission efficiency^30^.

## Example of an autonomous AI: IDx-DR

The management of a person with diabetes requires regular diabetic eye exams to prevent blindness and vision loss^31^. The patient is typically referred by their primary care physician to an eye care provider for an in-person diabetic eye exam, generally requiring a separate clinic appointment. With implementation of autonomous AI at the point-of-care for the diabetic eye exam, screening for diabetic retinopathy can be performed during the same primary care encounter. Autonomous AI for the diabetic eye exam has been validated against patient outcome, with high sensitivity and specificity for diagnosing diabetic retinopathy at the point of care, and has been shown to be cost-effective from the patient perspective^19,32^. Developed under a rigorous ethical framework^16^, with FDA clearance of the first autonomous AI in 2018^33^, specifically for the diabetic eye exam (IDx-DR, Digital Diagnostics, Coralville, IA)^34^, the Centers for Medicare and Medicaid Services (CMS) recently confirmed national coverage and reimbursement for such autonomous AI^34,35,34^.

In our analysis, we considered that the autonomous AI in this case may potentially reduce GHG emissions, by avoiding both those related to patient travel to the eye care provider, as well as GHG emissions generated by the healthcare facility where the in-person exam would take place.

## Methodology

In our analysis, we compare the marginal contribution, i.e., the additional GHG emissions of a single additional diabetic eye exam, either through the traditional eye care provider, or the autonomous AI, at the margin. Thus, our comparison does not consider emissions related to the care episode that leads to the diabetic eye exam, emissions arising from the design and development (including training) of the AI system, or the emissions resulting from the education and training of the ophthalmologist. We compare the marginal contributions, to CO_2_-eq emissions only, of a diabetic eye exam for a single patient, performed at the point of care by the autonomous AI for the diabetic eye exam, to the estimated marginal emissions if this diabetic eye exam is instead performed by an ophthalmologist after referral. We use the Environmental Protection Agency (EPA) in the U.S., conversion table, based on the present electricity generation mix, to convert a kWh of energy results in carbon emissions of 7.09 × 10^−4^ metric tons CO_2_-eq, or 0.709 g CO_2_-eq per Wh of energy^36^.

We set the marginal GHG emissions for a diabetic eye exam by an ophthalmologist, after referral, to that estimated by Dacones et al. for an in-person outpatient visit, i.e., ~8 × 10^3^ g CO_2_-eq per patient encounter^25^.

In our comparison, when calculating the marginal contribution of the autonomous AI we account for the fact that a ‘disease present’ output will lead to an in-person referral with an eye care provider—in our example about 20% of all cases will lead to a referral, with a concomitant increase in GHG emissions^34^.

## Results

This autonomous AI systems’ diagnostic computations, from interactive image capture, through AI inference—the AI is completely locked and deterministic after FDA authorization—and diagnostic output to the electronic health record and provider, occupies about 144 × 10^9^ CPU cycles. As an example, computations run for 10 s (0.003 h) on 4 parallelized Nvidia Tesla K80 processors^37^. AI power consumption estimates vary in the literature. For machine learning inference heavy applications, estimates range, for a single CPU, between 2.1 and 25 Wh. Using Sommer et al.’s estimate of 2.1 Wh^38^, and Bergman et al.’s estimate of 3.3 KWh/24 h^39^, respectively, we estimate power usage for a single autonomous AI diagnostic exam to be between 0.03 and 0.3 Wh.

Under these assumptions, each autonomous AI diagnostic exam generates 0.02–0.2 g CO_2_-eq emissions per exam. As mentioned above, if we account for 20% of patients receiving a diagnostic output requiring an additional in-person diabetic eye exam^34^, the total emissions are estimated to be 1.6 × 10^3^ g CO_2_-eq leading to an 80% reduction if patients get their initial diabetic eye exam using autonomous AI as opposed to in-person diabetic eye exams.

## Discussion

The use of a specific autonomous AI can lead to an 80% reduction in GHG emissions in the present state. We only compared the marginal GHG emissions generated by one additional diabetic eye exam, and did not consider training of either the autonomous AI system or the eye care provider, and other personnel involved in the in-person visit. If we were to do so, the comparison would be more stark: a retina specialist performing the in-person diabetic eye exam typically undergoes 8–10 years of training. Annual CO_2_-eq emissions per year of higher education per student for the US are estimated at 5 × 10^6 ^g CO_2_-eq, or a total of 5 × 10^7 ^g CO_2_-eq for the entire training^40^. Such a specialist may perform 100,000 diabetic eye exams in their career^16^, so that training may contribute 0.5 × 10^3 ^g CO_2_-eq to GHG emissions per exam. Training the AI may take 5 × 10^8^ g CO_2_-eq, as mentioned above. Such an autonomous AI system has almost unlimited scalability, so that it may perform diabetic eye exams for 30 × 10^6^ people with diabetes. Assuming such an autonomous AI functions without retraining for 5 years, the GHG emissions for the AI system training amounts to only 3 g CO_2_-eq/exam, in this worst-case scenario, <1% of the training for the human specialist. However, because of the many additional assumptions necessary in accounting for training, we only considered any marginal reduction in greenhouse emissions—and assume GHG emissions for eye care provider education and training and autonomous AI training are accounted for.

Limitations to this study include that we used 2021 estimates for all variables. These are subject to change, for example the production mix of power generation is expected to change over the coming years due to various initiatives, and thereby the GHG emissions per kWh will also change. The carbon efficiency of computer hardware is also changing, and thus the power consumption per inference, all else being equal, will change as well.

Based on the above assumptions and limitations, and extrapolation of currently available data estimates from 2021, autonomous AI has the potential to substantially lower healthcare GHG emissions, and thereby compensate for increasing carbon emissions attributed to information technology^41^. As use of autonomous AI systems expand in the healthcare industry, measurement of real-world carbon emissions attributed to these systems in comparison to usual care will help elucidate the potential contributions of autonomous AI in reducing healthcare emissions.

## Data Availability

Data utilized in this manuscript are available without restriction from their respective references.
